# Mechanisms of Hyperhomocysteinemia Induced Skeletal Muscle Myopathy after Ischemia in the CBS^−/+^ Mouse Model

**DOI:** 10.3390/ijms16011252

**Published:** 2015-01-06

**Authors:** Sudhakar Veeranki, Suresh C. Tyagi

**Affiliations:** Department of Physiology & Biophysics, University of Louisville, Louisville, KY 40202, USA; E-Mail: s0tyag01@exchange.louisville.edu

**Keywords:** homocysteine, skeletal muscle, PGC-1α, PPARγ, nitrotyrosylation, ischemia, CBS, CSE, MTHFR, H_2_S

## Abstract

Although hyperhomocysteinemia (HHcy) elicits lower than normal body weights and skeletal muscle weakness, the mechanisms remain unclear. Despite the fact that HHcy-mediated enhancement in ROS and consequent damage to regulators of different cellular processes is relatively well established in other organs, the nature of such events is unknown in skeletal muscles. Previously, we reported that HHcy attenuation of PGC-1α and HIF-1α levels enhanced the likelihood of muscle atrophy and declined function after ischemia. In the current study, we examined muscle levels of homocysteine (Hcy) metabolizing enzymes, anti-oxidant capacity and focused on protein modifications that might compromise PGC-1α function during ischemic angiogenesis. Although skeletal muscles express the key enzyme (MTHFR) that participates in re-methylation of Hcy into methionine, lack of trans-sulfuration enzymes (CBS and CSE) make skeletal muscles more susceptible to the HHcy-induced myopathy. Our study indicates that elevated Hcy levels in the CBS^−/+^ mouse skeletal muscles caused diminished anti-oxidant capacity and contributed to enhanced total protein as well as PGC-1α specific nitrotyrosylation after ischemia. Furthermore, in the presence of NO donor SNP, either homocysteine (Hcy) or its cyclized version, Hcy thiolactone, not only increased PGC-1α specific protein nitrotyrosylation but also reduced its association with PPARγ in C2C12 cells. Altogether these results suggest that HHcy exerts its myopathic effects via reduction of the PGC-1/PPARγ axis after ischemia.

## 1. Introduction

The metabolic disorder HHcy is an independent risk factor for vascular disease [1] and also affects other organ systems in both human and animal models [2,3,4,5,6,7,8,9,10]. It has been suggested that HHcy also causes skeletal muscle injury and elderly frailty [11]. The CBS^−/+^ mice, an animal model of HHcy, also exhibit lower than normal body weight when compared to age-matched normal wild type (WT) mice [12]. However, mechanisms for such muscle weakness and reduced body weight remain unknown.

HHcy results from accumulation of the non-protein coding sulfur-containing amino acid, homocysteine, in plasma [11]. Genetic factors (gene mutations), nutritional imbalance, age, sex, physical activity and disease states such as diabetes and chronic renal failure were shown to modulate homocysteine levels [11,13,14,15]. Mutations in the key Hcy-metabolizing enzymes such as MTHFR and CBS have been reported [11] in causing HHcy. The transsulfuration enzymes CBS and CSE are critical in that they not only metabolize Hcy irreversibly into cysteine, but also generate H_2_S. Another H_2_S producing enzyme, 3MST has also been reported [16]. Given that H_2_S acts as an anti-oxidant, expression levels of H_2_S producing enzymes (CBS, CSE and 3MST) dictate anti-oxidant capacity of tissues. However, the expression levels of these enzymes that determine the HHcy tolerance capacity and H_2_S production capacity in mouse skeletal muscles have not been characterized. Despite the fact that HHcy-mediated enhancement in ROS and consequent damage to regulators of different cellular processes is relatively well established in other organs [17,18], the nature of such events is unknown in mouse skeletal muscles.

Recent evidence suggested that HHcy attenuates ischemic skeletal muscle responses and compromises collateral angiogenesis through decline in PGC-1α function [19]. PGC-1α is an important transcriptional co-factor for PPARγ and has been shown to regulate both exercise capacity and angiogenesis [20,21]. In addition, disease conditions that attenuate skeletal muscles’ ability to upregulate PGC-1α have been reported [22], underscoring the significance of PGC-1α function. Furthermore, it has been demonstrated that increased PGC-1α expression was able to counter FOXO-mediated atrogene transcription and thereby plays a protective role in skeletal muscles [23].

Besides protein expression levels, post-translational protein modifications constitute important regulatory mechanisms that govern various cellular events including ischemic responses. Critical protein interactions are often determined by post-translational protein modifications. For example, PGC-1α SUMOylation enhances association with its co-repressor, and prevention of SUMOylation enhances PPARγ-dependent transcription [24]. Likewise, phosphorylation, the very common regulatory post-translational modification that either inhibits or augments PGC-1α function, has been reported [25,26]. These findings and others underscore the significance of post-translational PGC-1α modifications in regulation of its association-dependent function, and the ability of various metabolic and physiological factors in controlling PGC-1α-dependent responses [24]. Although we showed that Hyperhomocysteinemia (HHcy) during the course of ischemia attenuates the expression of PGC-1α [19], whether HHcy also mediates PGC-1α specific protein modifications is unclear.

In the current study, we examined the anti-oxidant status of skeletal muscles and focused on protein modifications that might compromise PGC-1α function during ischemic angiogenesis. Although skeletal muscles express the key enzyme (MTHFR) that participate in re-methylation of Hcy into methionine, lack of trans-sulfuration enzymes (CBS and CSE) make skeletal muscles more susceptible to the HHcy-induced myopathy. Our study further indicates that elevated Hcy levels in the CBS^−/+^ mouse skeletal muscles caused diminished anti-oxidant capacity and contributed to enhanced total protein as well as PGC-1α specific nitrotyrosylation after ischemia. Furthermore, in the presence of NO donor SNP, either homocysteine (Hcy) or its cyclized version, Hcy thiolactone, not only increased PGC-1α specific protein nitrotyrosylation but also reduced its association with PPARγ in C2C12 cells. Altogether these results suggest that HHcy exerts its myopathic effects via reduction of the PGC-1α/PPARγ axis after ischemia.

## 2. Results

### 2.1. Skeletal Muscles Lack Hcy Trans-Sulfuration Enzymes

**Figure 1:**
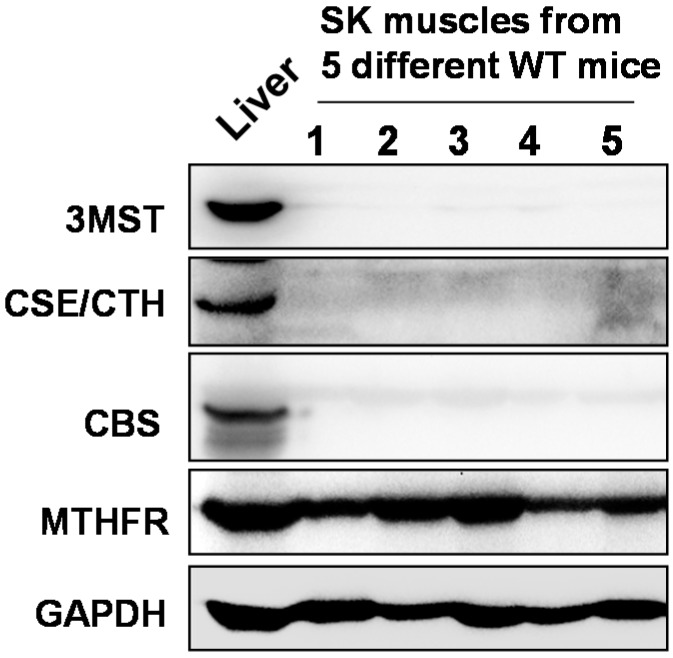
Western blot images showing the expression levels of various key Hcy metabolizing enzymes in normal thigh muscles: Methylenetetrahydrofolate reductase (MTHFR), Cystathionine β-synthase (CBS) and Cystathionine γ-lyase (CSE). We also measured the levels of another key H_2_S producing enzyme 3-mercaptopyruvate sulfur transferase (3MST) in conjunction with CBS and CSE in the thigh muscles. GAPDH was used as a loading control. Liver tissue lysate from the wild type mouse was used as a positive control.

To assess skeletal muscle capacity to effectively metabolize and remove Hcy from the system and to know the level of skeletal muscle susceptibility to toxic effects of HHcy, we have determined protein expression levels of various key Hcy metabolizing enzymes in the mouse thigh skeletal muscles. As shown in the Figure 1, when compared to the liver, WT skeletal muscles lack key trans-sulfuration enzymes CBS and CSE in the protein lysates. However, levels of MTHFR, a key enzyme in remethylation of Hcy into methionine, were detectable, albeit to a lesser extent when compared to that of liver tissue. We have also assessed the protein levels of another key H_2_S producing enzyme “3-mercaptopyruvate sulfur transferase” (3MST) and found that the levels were in the undetectable range. These findings suggest that mouse skeletal muscles are not only more susceptible to the HHcy-inflicted injury as they lack Hcy trans-sulfuration process, but also could not produce H_2_S, a known anti-oxidant.

### 2.2. Attenuated Skeletal Muscle Anti-Oxidant Capacity during HHcy

**Figure 2:**
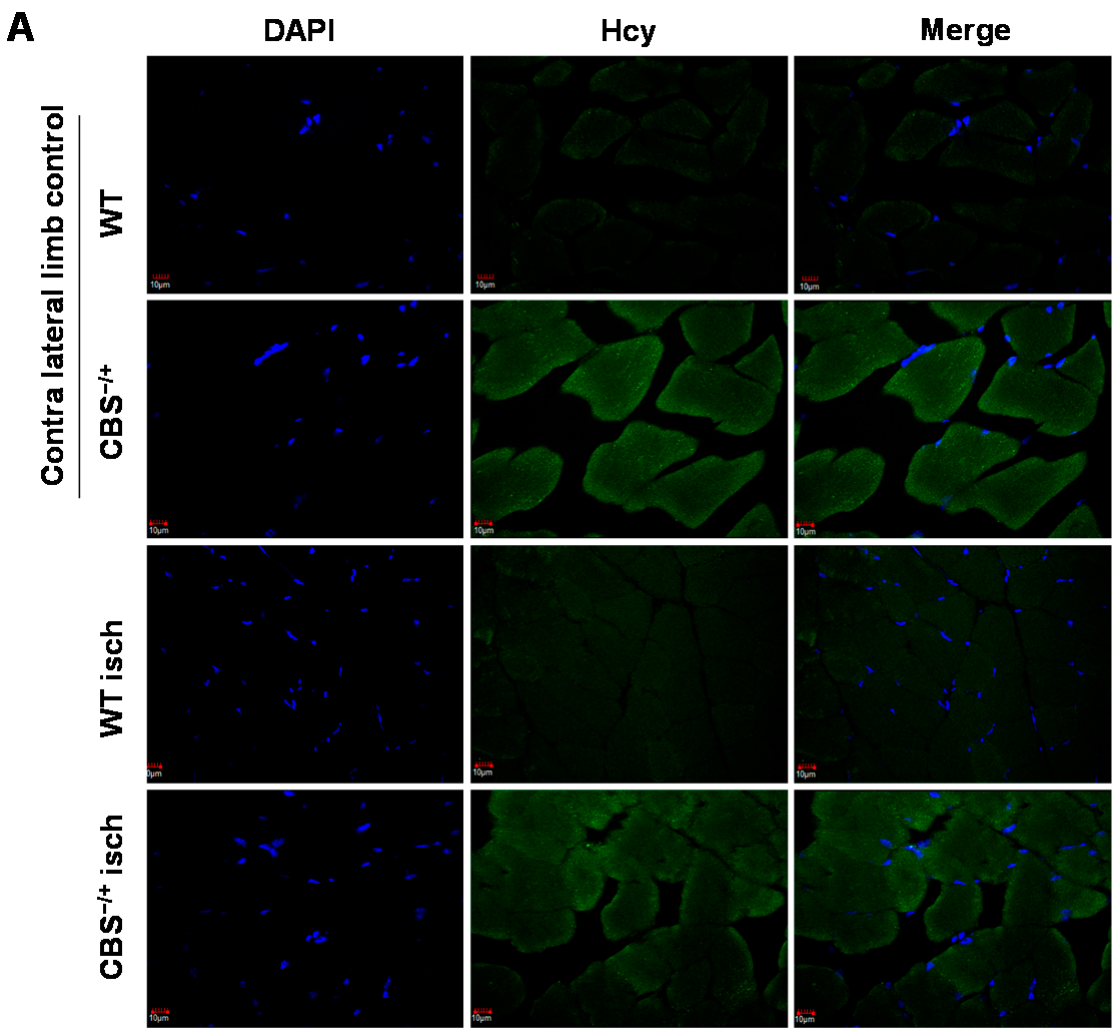

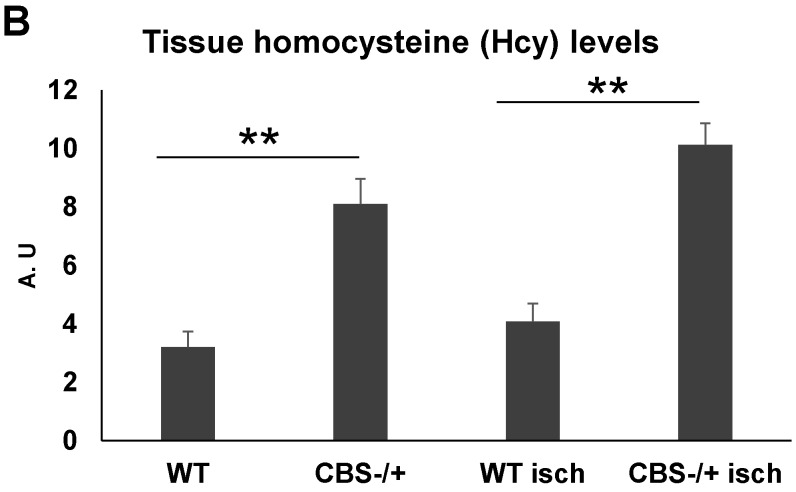
Elevated homocysteine presence in CBS^−/+^ mouse skeletal muscles. (**A**) Representative confocal images were obtained from WT and CBS^−/+^ mouse normal and ischemic gastrocnemius tissue sections. Blue fluorescence represents nuclei and green fluorescence represents homocysteine (Hcy) levels and (**B**) ImageJ quantification of Hcy levels from the confocal images of three different mice are presented in the bar graph. ** indicates *p* < 0.01. Scale bar: 10 µm.

**Figure 3:**
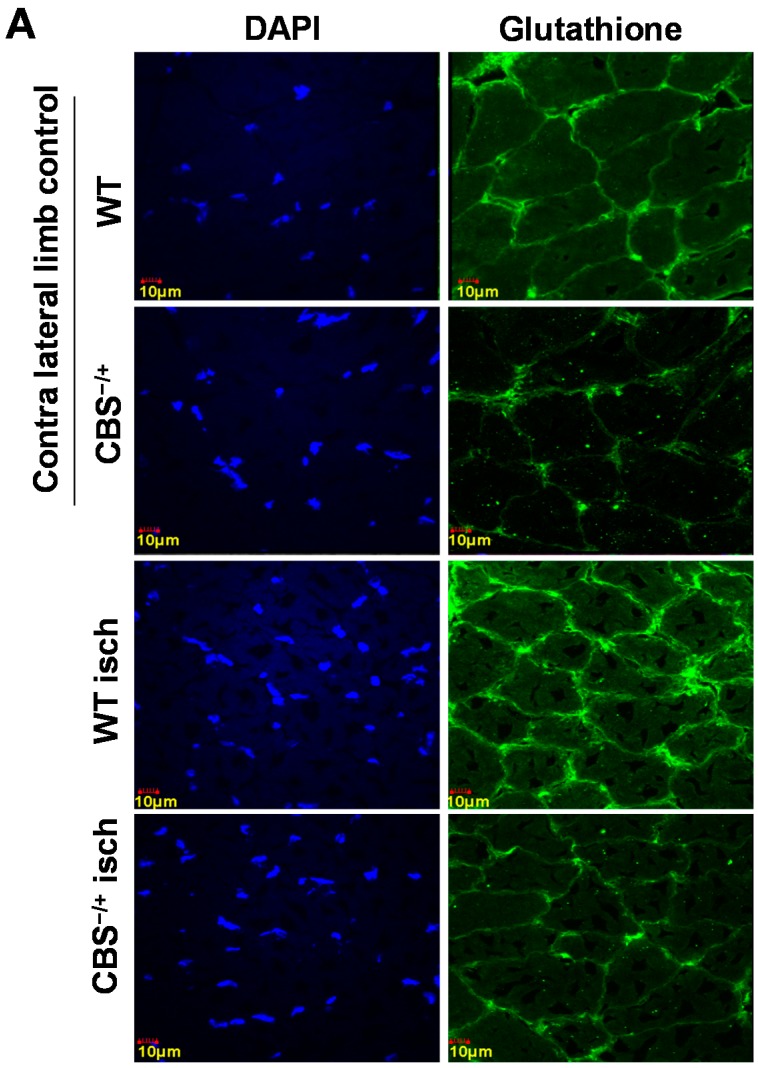

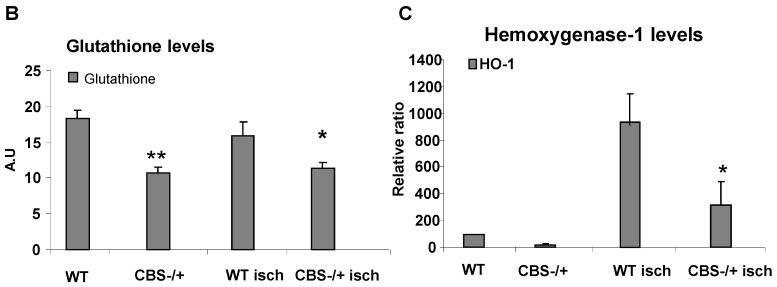
Attenuated anti-oxidant capacity in CBS^−/+^ mice. (**A**) Representative confocal images were obtained from the normal and ischemic gastrocnemius tissue sections of WT and CBS^−/+^ mice. Blue fluorescence represents nuclei and green fluorescence represents glutathione levels; (**B**) ImageJ quantification of glutathione levels from the confocal images obtained from three different mice is presented in the bar graph. ** indicates *p* < 0.01, and * indicates *p* < 0.05 and (**C**) Q-PCR data showing the levels of hemoxygenase-1 mRNA in normal and ischemic muscle tissue of WT and CBS^−/+^ mice is presented in the bar graph. * indicates *p* < 0.05.

To determine the levels of homocysteine in WT and CBS^−/+^ mouse skeletal muscles before and after ischemia, we performed immunohistochemical staining using the anti-Hcy (homocysteine) antibody. As observed in Figure 2A,B, CBS^−/+^ skeletal muscles exhibited relatively higher levels of Hcy. Next, to test if the anti-oxidant capacity in skeletal muscles is compromised during HHcy in addition to lack of H_2_S (a known anti-oxidant) production capability, we first enumerated the levels of key anti-oxidant glutathione in normal as well as ischemic skeletal muscle sections. As depicted in Figure 3A,B, the glutathione levels were significantly attenuated in both the normal and ischemic CBS^−/+^ mouse tissue sections when compared to that of the WT muscle sections. In addition, we also determined the levels of another key anti-oxidant enzyme Hemoxygenase-1 (HO-1) in the same set of tissue samples through Q-PCR. As presented in Figure 3C, the levels of HO-1 are not significantly different in normal tissue sections between WT and CBS^−/+^ mice. However, the HO-1 level induction was significantly decreased after ischemia in CBS^−/+^ skeletal muscles when compared to that of the WT muscles. Together, all these results indicate a heightened propensity for ROS accumulation, especially during ischemic conditions.

### 2.3. Enhanced Protein Nitrotyrosylation in Ischemic Skeletal Muscles during HHcy

**Figure 4:**
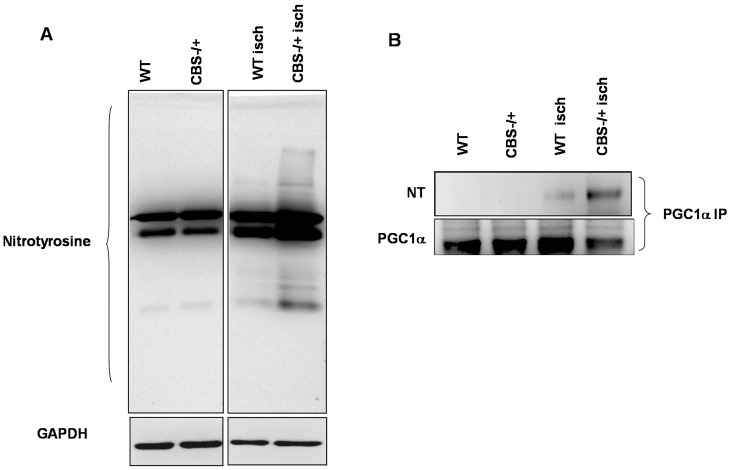
Enhanced protein nitrotyrosine levels in CBS^−/+^ mice during ischemia. (**A**) Western blot imaging showing the total protein nitrotyrosine levels from the normal and ischemic muscle tissue lysates of WT and CBS^−/+^ mice. GAPDH was used as a loading control and (**B**) Eluates from the PGC-1α immunoprecipitation from the same lysates as above were resolved on western blots and then probed with the nitrotyrosine and PGC-1α specific antibodies.

To find if there are any changes in post-translational protein modifications consequent to the attenuated anti-oxidant capacity during HHcy, we first looked at the protein nitrotyrosine levels in whole protein lysates of normal and ischemic tissues of WT and CBS^−/+^ mice. The results (Figure 4A) suggest that during HHcy after ischemia there was enhancement in the total protein nitrotyrosylation. To further know specifically if PGC-1α, an important regulator of exercise capacity and angiogenesis, was also modified by protein nitrotyrosylation, we assessed the nitrotyrosine levels after pull-down of PGC-1α from the total protein levels. As shown in Figure 4B, relatively higher levels of protein nitrotyrosine on PGC-1α were found in ischemic samples of CBS^−/+^ mouse skeletal muscles.

### 2.4. Inhibition of PGC-1α Interaction with PPARγ in the Presence of Hcy and NO Donor

**Figure 5:**
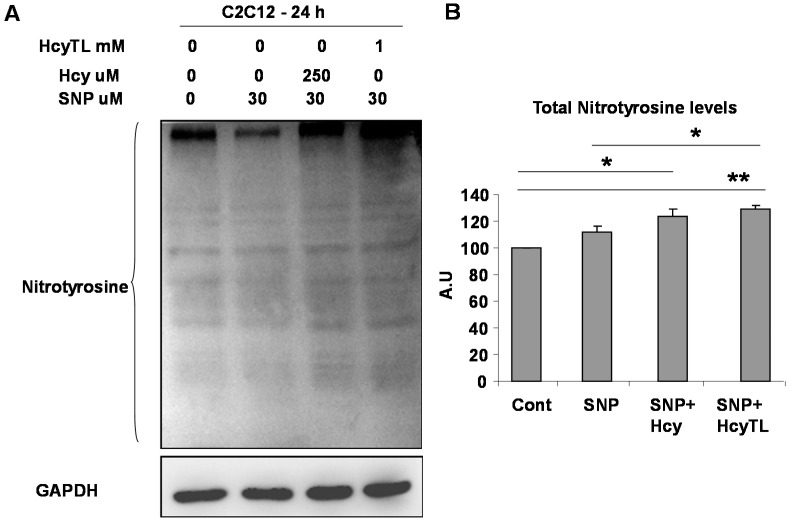
Hcy or its metabolite HCyTL increases protein nitrotyrosylation in the presence of nitric oxide (NO) donor sodium nitroprusside (SNP) in C2C12 cells. (**A**) A representative western blot is presented. Total protein lysates from the treated C2C12 cell lysates were resolved on SDS-PAGE gel and were probed with the anti-nitrotyrosine antibody. GAPDH was used as a loading control and (**B**) Quantification of total protein nitrotyrosylation levels from the treated C2C12 cells from three different experiments are depicted in the bar graph. * indicates *p* < 0.05 and ** indicates *p* < 0.01.

**Figure 6:**
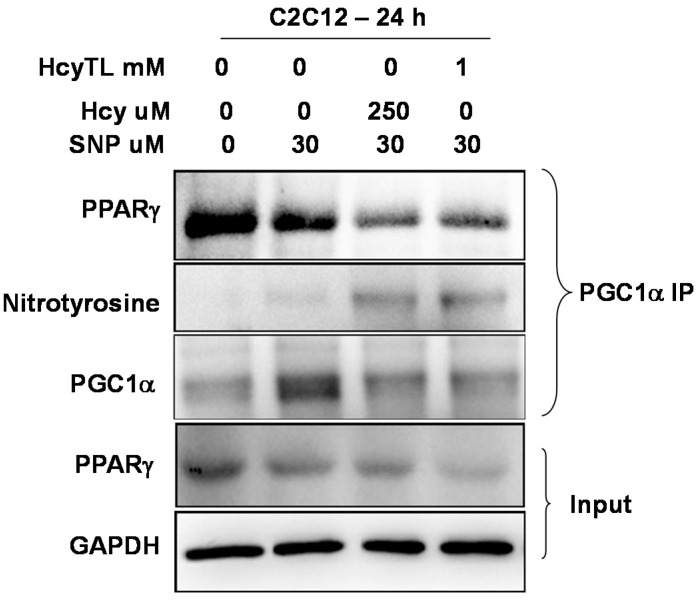
Western blot images showing the levels of PPARγ, nitrotyrosine and PGC-1α in the eluates of PGC-1specific immunoprecipitation from different treatment groups of C2C12 cell lysates. GAPDH indicates input levels for the immune-precipitation experiments across the groups. The levels of PPARγ were also probed in the total lysates and are not significantly different (data not shown).

To further understand the consequences of PGC-1α protein nitrotyrosylation and the conditions that favor protein nitrotyrosylation, we used the *in vitro* C2C12 myoblast model cell line. Previous study showed that NO donor SNP is toxic to C2C12 cells [27]. In light of this finding, we used a dose of SNP (30 μM) that is non-toxic to the cells in a 24 h period. All of our treatments did not produce any significant change in the cell morphology of differentiated C2C12 cells after the 24 h treatment period (data not shown). Differentiated C2C12 cells were treated with homocysteine or its cyclized metabolite homocysteine thiolactone (HcyTL) in the presence of nitric oxide donor SNP for 24 h. Cell lysates were assessed for total protein nitrotyrosine levels, as well as specific protein nitrotyrosine levels on PGC-1α. As show in Figure 5A,B, there was relatively increased nitrotyrosylation after Hcy or HcyTL treatment in the presence of NO donor SNP. Furthermore, there were increased nitrotyrosine levels on immunoprecipitated PGC-1α upon Hcy or HcyTL treatment in the presence of NO donor SNP (Figure 6). In addition, apparently there was an inverse relation between the associated PPARγ and the level of nitrotyrosylation present on the PGC-1α (Figure 6) after the PGC-1α specific pull-down. Given that the treatments of C2C12 cells did not significantly alter levels of PPARγ (Figure 6), reduced PPARγ-mediated downstream gene expression (as measured earlier for VEGF, [19]) coupled with its reduced association with PGC-1γ, together indicates that HHcy exerts its myopathic effects via reduction of the PGC-1α/PPARγ axis after ischemia through enhanced protein nitrotyrosylation.

## 3. Discussion

The Hcy trans-sulfuration enzymes, CBS and CSE not only covert Hcy into cysteine and help in irreversible removal of Hcy, but also produce H_2_S. Lack of expression of these key enzymes makes skeletal muscles more susceptible for myopathic effects of HHcy for the following reasons: (1) Hcy competes with the cysteine transporters [11] to get into the muscle fibers and during HHcy, homocysteine might decrease the effective local concentrations of cysteine and thereby promote oxidative stress, as cysteine is the precursor for anti-oxidant glutathione. Our measurements of glutathione levels (Figure 3A) and homocysteine (Hcy) (Figure 2) in CBS^−/+^ mouse tissue sections further support this phenomenon. In addition, reduced glutathione levels and increased oxidative stress has been reported recently in the skeletal muscles of rat model of HHcy [28]; (2) Lack of CBS, CSE and 3MST enzymes might lower the threshold of ROS-inflicted damage due to lack of known anti-oxidant H_2_S [29]; (3) HHcy causes alterations on the cellular proteins through protein nitrotyrosylation and might influence the levels of anti-oxidant enzymes such as SOD. Other reports also suggested similar protein modification in different tissues during HHcy [30]; (4) By decreasing the bioavailability of NO: previous studies showed that ROS increase results in decreased NO bioavailability by converting it into damaging peroxynitrite (ONOO−) radicals [31]. Increases in NO production and its protective role in ischemic tissues were observed in earlier studies [32,33]. Here we provide evidence for the attenuated anti-oxidant capacity in both normal and ischemic CBS^−/+^ skeletal muscle tissues; such decreases in the anti-oxidant capacity, in turn, lead to adverse protein nitrotyrosylation of key proteins, such as PGC-1α during ischemic injury and might potentially compromise the beneficial effects of NO and PGC-1α.

Our previous study indicated that during HHcy there was comprised ischemic collateral formation and attenuated endothelial proliferation. Moreover, we showed that there was reduced muscle specific expression of VEGF levels [19]. In the current study, we found that there was diminished anti-oxidant capacity during HHcy. Ischemic muscle specific levels of both the glutathione levels and the hemoxygenase-1 level induction were reduced when compared to that of the wild type ischemic muscle tissues. Furthermore, we demonstrated that there was enhanced protein nitrotyrosylation concomitant with declined anti-oxidant capacity. The results from the current study suggest that enhanced nitrotyrosylation on the PGC-1α, in CBS^−/+^ mice ischemic tissues, might adversely affect its association with PPARγ and might contribute to ischemic attenuation of VEGF levels in the skeletal muscles [19]. Our *in vitro* data from the C2C12 cell line further support this phenomenon and demonstrate that PGC-1γ nitrotyrosylation adversely affects its interaction with PPARγ under the permissible environment of increased NO production coupled with elevated Hcy levels.

**Figure 7:**
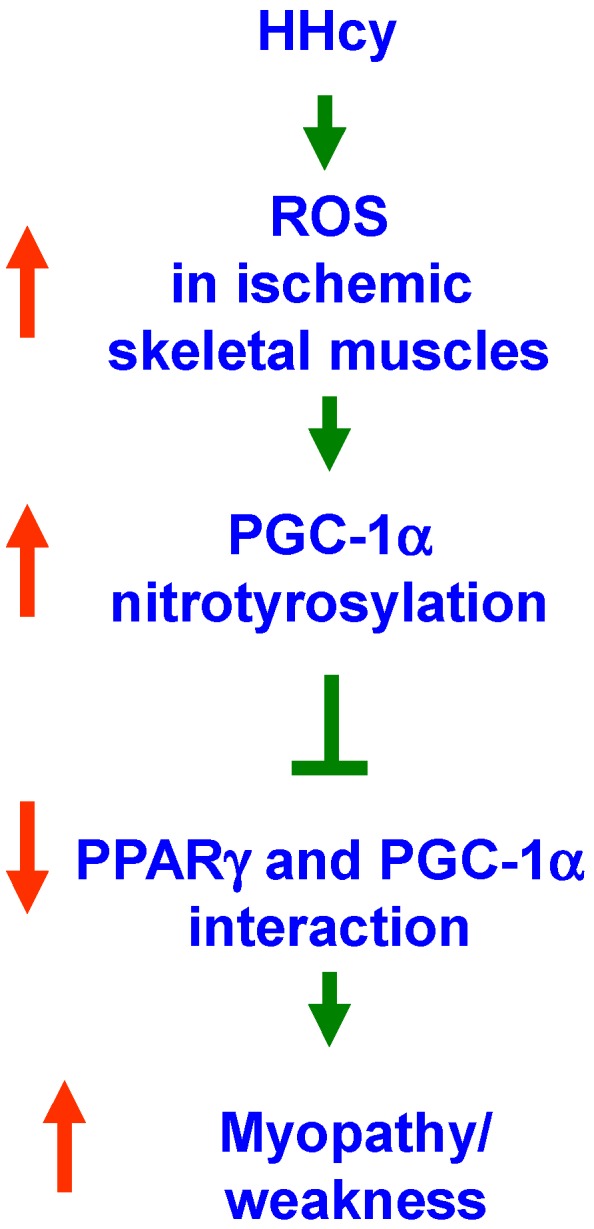
Summary model portraying connections between the various downstream events that lead to myopathy during HHcy.

We have summarized the current findings in a flow chart (Figure 7) to show the sequence of events that might lead to myopathy in the ischemic animals of HHcy. The relevance of these findings needs to be evaluated in human muscles with the HHcy condition. Currently the structural dynamics of nitrotyrosylation-mediated disruption of association between PGC-1α and PPARγ are not known. Future studies are necessary to unravel more insights in this regard. Though the *in vitro* Hcy concentrations used in the current study to treat C2C12 cells are higher in relative comparison to that of the plasma concentrations of CBS^−/+^mouse models [34], our findings are more relevant to the severe HHcy conditions (homocystinuria) as well as acute model of HHcy. No significant morphological changes were observed at concentrations (up to 250 μM) used for the 24 h treatment period, which further suggests that the higher Hcy treatment is well-tolerated by cells for a short duration.

## 4. Materials and Methods

### 4.1. Animal Care and Tissue Collection

WT (C57BL/6J) and CBS^−/+^ (B6.129P2-*Cbstm1Unc*/J 002853) mice were genotyped and reared till two months on regular chow and water as reported previously [19]. To avoid gender bias in our results, we used ~2 month old male mice in our experiments. The same hind limb ischemic muscle tissue samples were used for the current study to avoid unnecessary replicates as mentioned before [19]. All the animal studies were approved by the institutional IACUC (code: 11054, date: 9 July 2011) and are in conformity with the prescribed institutional standards.

### 4.2. Cell Culture

C2C12 cells were grown using DMEM medium with 10% FBS and 1% penicillin and streptomycin solution. At 80% confluence, cells were subjected to differentiation using DMEM medium containing 2% horse serum and 1% penicillin and streptomycin solution. After five days of differentiation, cells were treated with sodium nitroprusside (SNP) (30 μM) Hcy (250 μM) and Hcy thiolactone (1 mM) (Sigma-Aldrich, St. Louis, MO, USA) for 24 h as indicated. The growth medium was prepared using DMEM from the Life-Technologies (Grand Island, NY, USA), and for differentiation, we used DMEM medium from the ATCC (Manassas, VA, USA).

### 4.3. Immunoprecipitation

Equal amounts of lysates were incubated with the PGC-1α (Abcam, Cambridge, MA, USA) antibody and protein A/G plus agarose beads (Santa Cruz, Paso Robles, CA, USA) overnight. After appropriate washes, the beads were subjected to boiling for 5 min in the presence of Lamelli loading buffer containing BME (β-mercaptoethanol). The eluates were collected and were resolved on the SDS-PAGE gels.

### 4.4. Real Time PCR

Total RNA was isolated from the samples and quality and quantity were assessed using a spectro-photometer (NanoDrop, Wilmington, DE, USA). Total cDNA was synthesized using the Promega kit (Improm-II RT system, A3800, Promega, Madison, WI, USA) following the manufacturer’s instructions. The following primers were used to amplify the mRNA of interest using FastStart Essential DNA Green Master (Roche, Nutley, NJ, USA), 06402712001 cyber green chemistry: HO1F1 (5'–3'): AAGCCGAGAATGCTGAGTTCA, HO1R1 (5'–3'): GCCGTGTAGATATGGTACAAGGA, GAPDH F1: GAPDH-F1 (5'–3'): AGGTCGGTGTGAACGGATTTG GAPDH-R1 (5'–3'): TGTAGACCATGTAGTTGAGGTCA. Data was analyzed by calculating normalized relative ratios using c_q_ values.

### 4.5. Western Blotting

Tissues were homogenized and lysed with the RIPA lysis buffer containing protease and phosphatase inhibitors as described earlier [19]. After treatment, cells were lysed with the buffer and sonicated; the cleared supernatant was collected after centrifugation. Protein quantities across the samples were determined using Bradford reagent (Bio-Rad, Hercules, CA, USA). Equal quantities of protein samples were resolved using SDS-PAGE gel as described before [19]. After probing the membranes with primary and secondary antibodies along with appropriate washes, chemiluminescence signal was detected using the Bio-Rad ChemiDoc™ XRS+ System and Image Lab™ Software (Bio-Rad). For quantification, we used lysates from three different samples.

### 4.6. Antibodies

The antibody sources are: Anti-PPARγ (sc-7273),Anti*-*PGC-1α (sc-13067), Anti-CBS (sc-67154), Anti-CSE (sc-374249), Anti-3MST (sc-374326), and Anti-Nitrotyrosine (SC-32731) are from Santa Cruz, (Paso Robles, CA, USA); Anti*-*PGC-1α (ab-54481), Anti-MTHFR (ab-55530), Anti-Glutathione (ab-19534) and Anti-Hcy (ab-15154) are from Abcam (Cambridge, MA, USA); and Anti*-*GAPDH (MAB374) is from Millipore (Billerica, MA, USA). HRP-conjugated secondary antibodies are from Santa Cruz Biotechnology (Dallas, TX, USA) and Alexa Fluor-conjugated secondary antibodies are from Life Technologies (Grand Island, NY, USA).

### 4.7. Confocal Imaging

Ischemic skeletal muscle (gastrocnemius) tissues were used from the WT and CBS^−/+^ mice hind limbs after seven days of femoral artery ligation, as reported in our previous manuscript [19]. Briefly, tissue sections were fixed with 4% paraformaldehyde and incubated with appropriate primary and then secondary antibodies and then DAPI stain before mounting. Images were captured using a laser scanning confocal microscope (Olympus FluoView1000, Pittsburgh, PA, USA).

### 4.8. Statistical Analysis

Images from the western blotting were obtained and analyzed using the Image lab (Bio-Rad, Hercules, CA, USA). *p* value <0.05 was considered significant. The Student *t*-test was used to enumerate the levels of significance between the two different groups. Quantification of confocal image intensities was made using the ImageJ software.

## Abbreviation

PGC-1α: Peroxisome proliferator-activated receptor gamma coactivator 1
PPARγ: Peroxisome proliferator-activated receptor gamma
HHcy: hyperhomocysteinemia
Hcy: homocysteine
CBS: cystathionine beta-synthase
CSE: cystathionine γ-lyase
3MST: 3-mercaptopyruvate sulfurtransferase
MTHFR: methylenetetrahydrofolate reductase
ROS: Reactive oxygen species
HO-1: heme oxygenase-1
NO: nitric oxide
H_2_S: hydrogen sulfide
SNP: Sodium nitroprusside
